# The Role and Application of MAdCAM-1/α4β7-Induced Lymphocyte Migration in Inflammatory Enterohepatic Diseases

**DOI:** 10.3390/biomedicines13112659

**Published:** 2025-10-29

**Authors:** Chuchu Yu, Yuqing Pan, Aojie Mao, Yu Zhao, Qiaohong Liu, Yiyang Hu

**Affiliations:** 1 Institute of Liver Diseases, Shuguang Hospital Affiliated to Shanghai University of Traditional Chinese Medicine, Shanghai 201203, China; 2 Key Laboratory of Liver and Kidney Diseases (Ministry of Education), Shanghai 201203, China; 3 Shanghai Clinical Key Laboratory of Traditional Chinese Medicine, Shanghai 201203, China; 4 Clinical Research Center, Shuguang Hospital Affiliated to Shanghai University of Traditional Chinese Medicine, Shanghai 201203, China

**Keywords:** cell adhesion molecules, mucosal address cell adhesion molecule 1, α4β7, lymphocyte migration, inflammatory enterohepatic diseases

## Abstract

Inflammation drives the development of multiple inflammatory enterohepatic diseases. The recruitment of immune cells to inflammatory tissues is essential for maintaining immune homeostasis, mediating immune responses and regulating inflammation. MAdCAM-1/α4β7 is a pair of homing ligand and receptor that plays important roles in lymphocyte migration. Their binding induces lymphocytes to cross endothelial structures into corresponding lymphoid tissues, contributing to the inflammatory response. Aberrant lymphocyte migration due to excessive binding is closely related to the occurrence and development of inflammatory bowel disease and liver inflammation. In this review, we focus on the activation of α4β7 and binding to MAdCAM-1 how to induce the migration of multiple kinds of lymphocytes. Additionally, we describe the intestinal microbiota and its metabolites associated with MAdCAM-1/α4β7 in inflammatory enterohepatic diseases. We also discuss the current status of the development of monoclonal antibodies and small molecule drugs targeting MAdCAM-1/α4β7 for the remission and treatment of inflammatory enterohepatic disease. Future research should focus on enhancing hepatic specificity and conducting well-designed clinical trials for inflammatory liver diseases to confirm therapeutic efficacy.

## 1. Introduction

Inflammation drives the development of multiple inflammatory gut-liver conditions. In fatty liver disease, primary sclerosing cholangitis, liver cirrhosis, and other liver diseases, the inflammatory process is an integral part of the progression of these diseases, in addition to their respective etiologies of fat accumulation, cholestasis, and fibrosis formation [1,2]. While the pathogenesis of inflammatory bowel disease (IBD) involves multiple factors including genetics, environment, immune responses, and gut microbiota [3], the central role of inflammation in driving the progression of both ulcerative colitis (UC) and Crohn’s disease (CD) is well-established. So, suppressing inflammation and controlling its progression is important in the treatment of these enterohepatic diseases.

Dysregulation of lymphocyte transport plays an important role in the inflammatory process in these diseases [4]. The ability of lymphocytes to selectively enter specific lymphoid tissues is known as “homing”, and integrins and their ligands play a key role among the various molecules responsible for regulating lymphocyte homing, a complex process of specific migration [5]. A central pathway is mediated by integrin α4β7 following its activation and binding to MAdCAM-1, which directs the migration of various lymphocyte populations, including T cells, whereas overexpression of MAdCAM-1 or excess lymphocyte homing induced by MAdCAM-1/α4β7 also drives the pathogenesis of intestinal inflammatory disorders, including IBD. Through gut-liver axis communication, these lymphocytes migrate to hepatic tissue, further inducing inflammatory responses that contribute to the pathogenesis of inflammatory liver diseases such as primary sclerosing cholangitis (PSC) and non-alcoholic steatohepatitis (NASH). This continuum of pathological events further confirms the intimate connection between the gut and the liver within the inflammatory immune mechanisms of inflammatory enterohepatic diseases. Given the pivotal role of the MAdCAM-1/α4β7 pathway in sustaining this gut-liver inflammatory axis, monoclonal antibodies targeting this pathway, such as Vedolizumab, have emerged as a key therapeutic strategy for IBD [6].

This paper is for reviewing and emphasizing the role of MAdCAM-1/α4β7 in lymphocyte migration and inflammatory enterohepatic diseases. In addition, we will highlight specific intestinal microbiota and their metabolites associated with the expression of MAdCAM-1/α4β7, as well as monoclonal antibody drugs and small molecule drugs targeting MAdCAM-1/α4β7. Understanding MAdCAM-1/α4β7-regulated lymphocyte migration could open new paths for the treatment of gut-liver inflammatory diseases.

## 2. Structure and Expression of MAdCAM-1/α4β7

**Figure 1:**
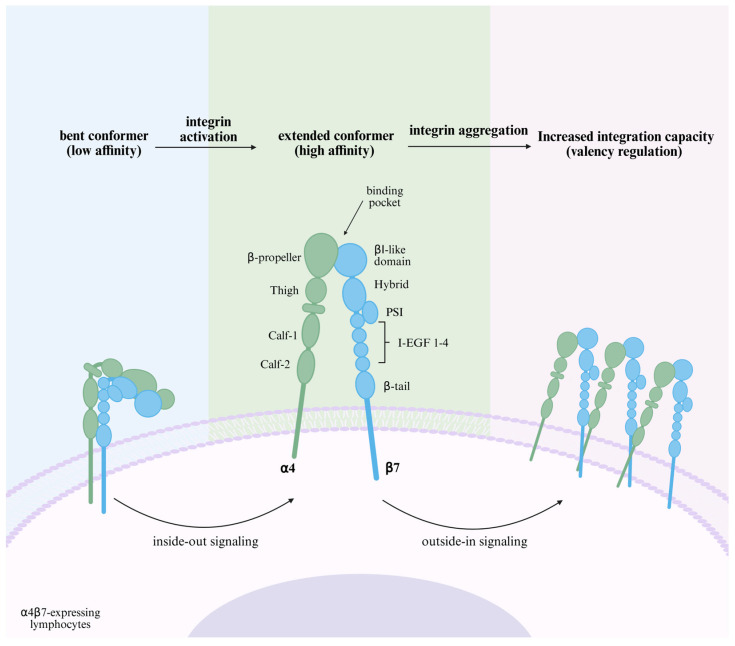
**Schematic structure and affinity regulation of α4β7.** α4β7 is bent in a quiescent state and the ligand-binding pocket is hidden from MAdCAM-1 binding. The “inside-out signaling” triggered by the binding of chemokines and their receptors induces conformational changes in the integrin structure, extending the domains and exposing the hidden ligand-binding pocket, which increases the affinity of α4β7 and begins to bind to MAdCAM-1. This ligand binding leads to integrin-mediated “outside-in signaling”, affecting cytoskeletal rearrangement, further aggregation of α4β7, and a change in ‘valency,’ which enhances adhesion strength [13]. Created in BioRender. Pan, Y. (2025) https://BioRender.com/hggjq78 (accessed on 6 March 2025).

Integrins are transmembrane heterodimers that act as major cell adhesion transmembrane receptors and 24 integrin heterodimers have been reported, consisting of 18 α-subunits and 8 β-subunits [7], each of which is a surface glycoprotein greater than 1600 amino acids in length, and consists of a short intracellular region, a helical transmembrane region, and a large extracellular region. The extracellular structural domain of each subunit serves as the ligand-binding site for the heterodimer [8]; the extracellular structural domain of α4 (180 kDa) contains: a hepta-bladed β-propeller, a thigh and two calf structural domains. It is worth noting that unlike other α-chains in the integrin family, α4 does not have an αI structural domain, and forms a binding pocket directly with the βI structural domain in the β-chain via the β-propeller structure. The extracellular structural domain of β7 (130 kDa) contains an insertion of heterodimeric βI structural domain, plexin-semaphorin- integrin (PSI), four cysteine-rich epidermal growth factor modules, and a β-tail structural domain (Figure 1) [9]. Integrins can be categorized into four types based on cell type or ligand specificity: leukocyte adhesion integrins, RGD (Arginine-Glycine-Aspartic Acid) -binding integrins, collagen-binding integrins, and laminin-binding integrins, of which leukocyte adhesion integrins are mainly involved in the regulation of inflammation [10]. α4β7 belongs to leukocyte adhesion integrins, which are expressed on a wide range of leukocytes, such as naïve and memory CD4+, CD8+ T cells, B cells, eosinophils, NK-cells, and a small number of monocytes [11]. The expression and binding affinity of α4β7 are enhanced by chemokines, including CXCL12, CCL12, and CCL25, with CXCL12 demonstrating the most pronounced effect [12].

**Figure 2:**
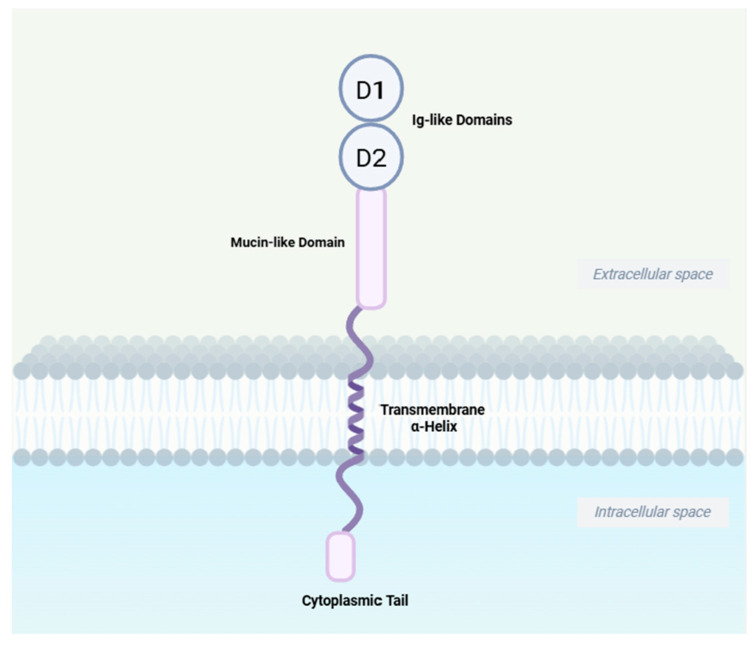
**Schematic structure of MAdCAM-1.** MAdCAM-1 contains two distal immunoglobulin-like structural domains, D1 and D2, which constitute the key structural elements for α4β7 binding; a mucin-like region which is the site of O-linked glycosylation and the L-selectin-binding domain; an α-helix transmembrane domain and a shorter cytoplasmic domain. Created in BioRender. Yu, C. (2025) https://BioRender.com/a5qc10v (accessed on 21 September 2025).

The interaction of integrins with the Immunoglobulin superfamily (IgSF) is particularly important during lymphocyte homing [14]. Mucosal address cell adhesion molecule 1 (MAdCAM-1), the ligand for α4β7, is a member of the IgSF family. Other members include intercellular adhesion molecule (ICAM), vascular cell adhesion molecule-1 (VCAM-1), and platelet/endothelial cell adhesion molecule-1 (PECAM-1), etc. [15,16]. MAdCAM-1 contains of two distal immunoglobulin-like structural domains, a mucin-like region, a transmembrane structural domain, and a cytoplasmic structural domain. It binds to integrin α4β7 via its immunoglobulin-like structural domain and to L-selectin through its mucin-like region when properly O-glycosylated (Figure 2) [15]. MAdCAM-1 is expressed on high endothelial venule (HEV) endothelial cells in gut-associated lymphoid tissues (GALT), as well as in the small intestine and colon of both healthy individuals and patients with IBD. Its expression is upregulated during active inflammation. While absent in normal liver tissues, MAdCAM-1 is expressed on portal vein endothelial cells and hepatic sinusoidal endothelial cells in inflammatory liver disease [12]. In humans and mice, its expression is induced by TNF-α, which is mediated through the NF-κB pathway [17,18].

## 3. Activation of α4β7 and Binding to MAdCAM-1 Induces Lymphocyte Migration

### 3.1. T Cell

**Figure 3:**
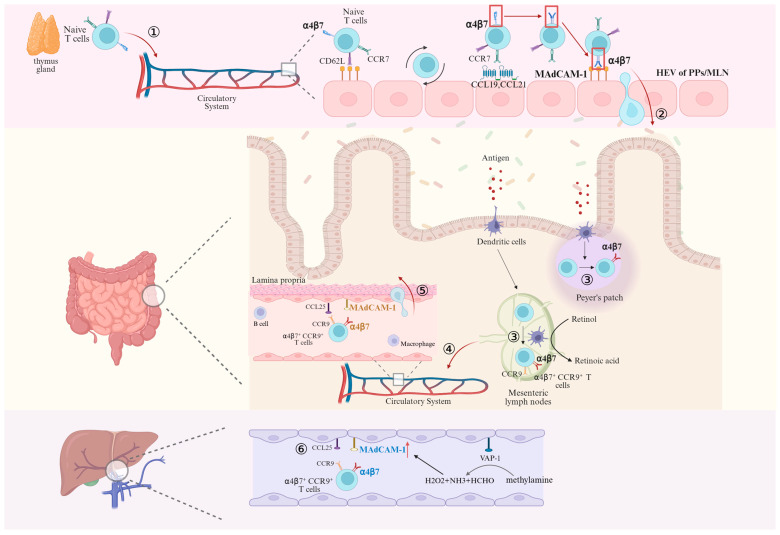
**Activation of α4β7 on naïve T cells and migration of naïve and mature T cells mediated by binding of α4β7 to MAdCAM-1.** ① After leaving the thymus, Naïve T lymphocytes enter the circulatory system and begin to slowly roll along endothelial cells through the “tethering” mainly mediated by L-selectin when passing through HEV. ② After activation of α4β7 mediated by CCR7, CCL19 and CCL21, the binding of α4β7 and MAdCAM-1 induces T cells to enter the Peyer’s patches (PPs) and mesenteric lymph nodes (MLN). ③ T cells encounter antigen-presenting dendritic cells in PPs and MLN that encode α4β7 expression in the presence of retinoic acid, differentiate into effector T cells highly expressing α4β7, and ④ subsequently return to the circulatory system via efferent lymphatics. ⑤ At this point, effector T cells that are highly expressing α4β7 can migrate into the lamina propria directly in binding to MAdCAM-1. ⑥ Effector T cells highly expressing α4β7 enter the liver parenchyma via hepatic sinusoidal endothelial cells in response to the inflammatory response after MAdCAM-1/α4β7 binding. Created in BioRender. Pan, Y. (2025) https://BioRender.com/2q5n9lo (accessed on 6 March 2025).

Naïve T cells released from the thymus need to enter secondary lymphoid organs to encounter their cognate antigens, at which point the naïve T cells express CD62L (L-selectin), C-C-chemokine receptor (CCR7), and low levels of α4β7 integrins [19]. However, because integrins are inactive (bent conformation) on resting circulating immune cells and do not readily interact with endothelial cells [20], the adhesion cascade begins with “rolling” mediated by “tethering”, where cells need to be pre-recruited to the canalicular wall and slowed down, which is mediated by selectins on lymphocytes and selectin ligands on the endothelium. In addition, the low-affinity linkage of α4β7 to MAdCAM-1 is involved in rolling. These interactions establish loose contacts between cells, which are repeatedly separated and re-established, resulting in leukocytes being able to have ample opportunity to sense chemokines while slowly rolling along the vessel wall [21]. These chemokines and G protein-coupled chemokine receptors form a rapid intracellular “inside-out” signaling [22] that induces conformational changes in integrins. These changes involve: the extension of the integrin’s extracellular region, the segregation of its subunits and transmembrane domains, and a rearrangement of the α-β chains in the area that binds ligands. Consequently, the integrin shifts from a compact, curved shape to an open one. This open conformation results in a higher binding affinity. When α4β7 binds to MAdCAM-1, cellular “outside-in” signaling occurs, resulting in a change in “valence” at the contact site, an increase in the number of receptor-ligand bonds [13], and a further strengthening of cellular adhesion, which allows T cells to enter into the intestinal lymphoid tissue via HEVs [23]. After entering the gut-associated lymphoid tissue and meeting with antigen-presenting dendritic cells, the dendritic cells convert retinol to retinoic acid by producing retinaldehyde dehydrogenase. The retinoic acid then activates the nuclear receptor, retinoic acid receptor (RAR), which forms a heterodimer with the retinoid X receptor (RXR) [24]. This complex then binds to retinoic acid response elements (RAREs) in the promoter regions of genes encoding α4β7 and CCR9, inducing their transcription in T lymphocytes and promoting the differentiation of naïve T cells into effector T cells with high α4β7 expression. These effector T cells subsequently return to the circulation via efferent lymphatics, and when once again following the circulation to the GALT, at which point they can be directly implanted in the lamina propria through the high affinity of α4β7 for MAdCAM-1 and the binding of CCR9 and CCL25 [12]. In addition, it has been reported that CCL25 induces a more extended active conformation of α4β7 by binding to CCR9, resulting in an increased affinity of α4β7 for MAdCAM-1 [25,26]. This process is summarized in Figure 3.

T cells isolated from the livers of mice with chronic DSS (Dextran Sulfate Sodium Salt) colitis, including both CD4+ and CD8+ T cells, expressed integrin α4β7 at higher levels than in control mice [27]. The transfer of intestinal pathogens beyond the mucosal barrier to the portal circulation and the liver allows MAdCAM-1, which is not expressed in the normal liver, to be expressed in the inflammatory environment. Lymphocyte recruitment requires not only MAdCAM-1/α4β7 interactions, but also specific chemokine signaling provided by CCL25, whose expression is usually restricted to the gut and thymus. However, it has been reported that CCR9+ (receptor for CCL25) cells make up to 20% of the lymphocytes that infiltrate the liver in PSC [28]. In summary, long-lived mucosal T cells are recruited to the liver in response to the abnormal expression of endothelial adhesion molecules and chemokines, which are typically restricted to the intestine [29]. Another evidence that α4β7+ T lymphocytes in the liver are initially activated in the intestine is that dendritic cells isolated from the liver or hepatic lymph nodes of patients with PSC fail to induce α4β7 and CCR9 expression in the absence of exogenous retinoic acid [30]. In contrast to the homing of lymphocytes in the intestines, although integrin α4β7 was detected on intestinal-activated CD8+ T cells in iFABP-OVA mice expressing ovalbumin (OVA) in small intestinal enterocytes and on hepatic-activated CD8+ T cells in TF-OVA mice expressing ovalbumin in hepatocytes, the co-expression of α4β7 and CCR9 was restricted to intestinal-activated CD8+ T cells, which exhibited a stronger migration potential. And this T-cell migration appeared to be unidirectional: gut-activated T cells were present in both the liver and intestine, whereas liver-activated T cells aggregated only in the liver [31]. Unlike homing in the gut, the low blood flow through the narrow vessels in the hepatic sinusoids means that T cells already have the opportunity to continuously interact with the endothelium, rendering the selectins that are important in intestinal homing redundant. However, the currently undefined process of “tethering” may still be necessary [32]. Furthermore, upregulation of the extracellular enzyme vascular adhesion protein-1 (VAP-1) during chronic inflammation induces MAdCAM-1 upregulation on the sinusoidal endothelium through deamination of methylamine (MA) in addition to directly supporting lymphocyte adhesion [33,34], as shown in Figure 3.

### 3.2. Migration of Lymphocytes Other than T Cells

#### 3.2.1. B Cell

B-lymphocyte homing appears to be controlled in a manner similar to that of T cells: naïve and memory B cells are recruited to GALT through the interaction of CD62L, CCR7, and α4β7 with CCL21 and MAdCAM-1 expressed on HEVs. Upregulation of intestinal homing markers, such as CCR9 and α4β7 integrins, on B-lymphocytes is mediated by dendritic cells that secrete retinoic acid (RA) [23]. Activated plasmablasts exhibited upregulated CCR9, CCR10, and α4β7, with α4β7 being critical for their intestinal homing. In β7^−/−^ mice, the number of IgA-secreting plasma cells in the lamina propria was reduced, and these mice were incapable of generating an IgA response, which impairs mucosal immunity [35]. Furthermore, the serum and intestinal levels of IgA following stimulation with T cell-dependent antigens were significantly lower in β7^−/−^ mice compared to wild-type mice [35,36]. Similarly, MAdCAM-1^−/−^ mice show a failure to generate an IgA response [37]. Unlike T-cell migration, the homing efficiency of B cells is lower than that of T cells because the level of L-selectin expression in B cells is more than 50% lower than that of T cells, despite the fact that B cells can be activated in HEV by two different chemokines and corresponding receptors (CCR7 and CCL21, CXCR4 and CXL12) [19,38]. Additionally, B cells, while capable of homing to the PPs to support mucosal immunity, are less efficient at returning to peripheral lymph nodes compared to T cells. Instead, B cells preferentially migrate to the spleen, whereas T cells primarily localize to the lymph nodes [39]. CD22 is more important for B-cell migration, but its absence does not affect T-cell migration [40].

#### 3.2.2. Eosinophil

Most studies on eosinophils have focused on rolling and adhesion mediated by the interaction of α4β1 with VCAM1 and fibronectin, but an important role of α4β7 for eosinophil recruitment into the gut under inflammatory conditions has also been reported: experimental gastrointestinal anergy induction in mice revealed that, eosinophil levels were significantly reduced in the colon of mice lacking β7 compared to wild-type mice [41]. In a clinical observation, vedolizumab treatment for one year led to improved global assessment scores and reduced eosinophil density in three of four eosinophilic gastrointestinal diseases (EGID) patients [42].

#### 3.2.3. Treg17 Cell

Treg17 cells, a unique subset of regulatory T cells producing IL-17, typically coexpress the canonical Treg marker Foxp3 alongside the Th17-characteristic cytokine IL-17A and transcription factor RORγt. The development of both Treg17 cells and their effector Th17 counterparts depends on the same lineage-specific master transcription factor, STAT3, reflecting a delicate balance between immune tolerance and inflammation [43]. Current research has moved beyond the simplistic definition of Foxp3+ IL-17+ cells and now aims to identify more specific surface marker combinations—such as the p-mTORʰⁱᵍʰ Treg17 population defined by Natarajan et al., which plays a protective role during mucosal infection [44]. Notably, administration of antibiotics resulted in down-regulation of MAdCAM-1 expression levels in the ileum, PPs and MLN, while Treg17 cells migrated out of the ileum to extra-intestinal tumor as well as to tumor-draining lymph nodes. Ectopic expression of MAdCAM-1 in mouse liver reduces the accumulation of α4β7+ Treg17 cells at tumor, thereby improving the therapeutic efficacy of immunotherapy [45].

#### 3.2.4. Non-Classical Monocytes

They express high levels of the CX3C chemokine receptor 1 (CX3CR1), which competitively inhibits CX3C chemokine ligand 1 (CX3CL1) and CCL3 from activating other, more destructive inflammatory cells, thereby protecting the endothelium and barrier system [46]. In dynamic adhesion assays, increased adhesion of peripheral blood monocytes from IBD patients to MAdCAM-1 was observed. Treatment with vedolizumab reduced monocyte adhesion to MAdCAM-1 to background levels. Further analysis revealed that the dynamic adhesion rate of non-classical monocytes to MAdCAM-1 was significantly higher than that of classical monocytes. Vedolizumab markedly reduced the adhesion rate of non-classical monocytes but had no significant effect on classical monocytes. Similarly, after transferring fluorescence-labeled classical and non-classical monocytes into the ileocolic artery of recipient mice, results showed that significantly more non-classical monocytes homed to the intestine compared to classical monocytes. Anti-α4β7 integrin treatment significantly reduced the accumulation of non-classical monocytes in the colon, with no notable effect on classical monocytes. These findings collectively indicate that α4β7 expression on non-classical monocytes plays a crucial role in mediating dynamic adhesion and gut homing in vivo [47].

#### 3.2.5. Innate Lymphoid Cell

Innate lymphoid cells (ILCs) do not express antigen-specific receptors; instead, they function primarily through the secretion of specific cytokines in a manner analogous to T cells, playing critical roles in host defense against pathogens [48]. Innate lymphoid cells (ILCs) types 1 and 3 may upregulate α4β7 via retinoic acid-mediated gut-specific imprinting thereby accomplishing the “homing receptor switch”. In contrast, expression of α4β7 on ILCs2 is acquired in a RA-independent manner in the bone marrow and ILCs2 can migrate directly to the intestine [49].

## 4. MAdCAM-1/α4β7-Induced Lymphocyte Homing Excess in Inflammatory Enterohepatic Disease

In addition to the anatomical homology of the liver and intestines originating from the endoderm, the theory of ‘intestine-liver axis’ proposed by Marshall in 1998 elaborated the mutual regulation and influence between the liver and intestine through the portal vein system, which was even more proved to have metabolic interactions and immune correlations. For example, impaired intestinal barrier function due to multiple causes allows bacterial metabolites to migrate and reach the liver through the portal vein, thereby exacerbating hepatic inflammation and leading to NASH, cirrhosis, and other diseases [50]. Whereas liver insufficiency can cause dysfunction in intestinal secretion, absorption, motility, barrier, and other aspects. For example, cirrhosis can lead to the formation of portal hypertension, which in turn can cause alterations in intestinal dynamics and dysbiosis of intestinal microbiota [51]. In addition to the gut-liver interconnection, activated immune cells disrupt tight junction proteins between epithelial cells, leading to increased intestinal permeability, and interfere with normal metabolic functions of hepatocytes in the liver, such as promoting lipid synthesis and inhibiting fatty acid β-oxidation. Factors released by inflammatory cells and damaged hepatocytes, including TGF-β, activate hepatic stellate cells, further aggravating disease progression. Thus, the synergistic interactions between immune and non-immune cells also play a critical role in the initiation and progression of inflammation [52,53].

### 4.1. Intestinal Diseases

In 1997, in attempting to determine whether MAdCAM-1 or its receptor α4β7 was a suitable target for therapeutic intervention in gut-associated inflammation, D’Picarella et al. found that β7- and MAdCAM-1-specific antibodies blocked lymphocyte recruitment to the colon in a colitis model, significantly reducing disease severity. This suggests that the interaction between MAdCAM-1 and α4β7 is closely related to the recruitment of lymphocytes to the intestine in chronic inflammatory diseases, and blocking their binding could be a relevant therapeutic target for inflammatory bowel disease patients [54]. In 2006, Christopher Bachmann et al. developed a novel intravascular ultrasound contrast agent targeting MAdCAM-1 to specifically detect and quantify intestinal inflammation in experimental ileitis. This agent provides a reliable, noninvasive method for diagnosing and monitoring intestinal inflammation [55]. Over the years, an increasing number of studies on inflammatory bowel disease have focused on this pathway. For example, angiotensin II type 1 receptor blockade can modulate TNF-α-induced MAdCAM-1 expression by inhibiting NF-κB nuclear translocation, thereby ameliorating colitis [17]. It was not until 2014 that the Food and Drug Administration (FDA) approved Vedolizumab for the treatment of adult patients with moderate to severe inflammatory bowel disease who have had an inadequate response, loss of response, or intolerance to one or more of the standard therapies (corticosteroids, immunomodulators, or tumor necrosis factor-alpha inhibitors) [56]. Moreover, a vaccination study in healthy volunteers showed that vedolizumab attenuated the response to enteral antigen challenge (oral cholera vaccine) without affecting the response to parenteral antigen challenge (intramuscular hepatitis B vaccine), indicating a high degree of gut-specific anti-inflammatory activity for Vedolizumab [57]. In addition to inflammatory bowel disease, a review of Vedolizumab for the treatment of non-inflammatory bowel disease-associated enteropathy demonstrated that Vedolizumab has clinical, endoscopic, and histologic improvements in patients with non-IBD-associated inflammatory bowel disease (e.g., microscopic colitis) as well as inflammatory disorders of the small bowel, including autoimmune enteropathy and common variable immunodeficiency-associated enteritis, suggesting that its role in the gut is not limited to inflammatory bowel disease [58].

### 4.2. Liver Diseases

#### 4.2.1. Primary Sclerosing Cholangitis

PSC is an inflammation of the intra- and extra-hepatic bile ducts leading to destruction of the bile duct epithelium and end-stage liver disease [59]. Approximately 70% of patients with PSC have or will develop concomitant IBD, with the majority of PSC-IBD patients suffering from ulcerative colitis (72%). Colon resection prior to the diagnosis of PSC reduces the risk of liver transplantation or death [59,60]. The significant association between PSC and IBD suggests that long-lived memory T lymphocytes expressing intestinal homing markers migrate to the liver via aberrantly expressed mucosal adhesion molecules and chemokines. Analysis of common T-cell receptor clonotypes in paired colon, liver, and blood samples revealed a higher degree of overlap between PSC-IBD samples than between normal intestinal and liver samples. This suggests that memory T-cells in the intestines and livers of PSC-IBD patients are reacting to common antigens [61]. Consistent with this, PSC is characterized by a peribiliary infiltrate composed mainly of T lymphocytes, a large proportion of which express the intestinal homing integrin α4β7 [62]. Several studies have also reported that MAdCAM-1 is aberrantly expressed on PSC hepatic endothelial cells in both large and small portal veins [63,64,65]. For example, the proportion of endothelial cells staining positivity for MAdCAM-1 calculated by H score was significantly higher in liver tissues of patients with long-term PSC (PSC-IBDLT). The proportion of β7-positive T cells was also significantly higher than in short-term patients and controls, with a positive correlation between disease duration and Itgb7 expression. The enzymatic activity of VAP-1 was higher in liver and intestinal tissues of PSC patients compared to controls, promoting the adhesion of intestinal-derived lymphocytes to hepatic endothelial cells in a substrate-dependent manner [34]. Seidel, D. et al. also found that activated CD8+ T cells in mouse GALT induce immune-mediated cholangitis in an antigen (OVA)-dependent manner [27].

#### 4.2.2. Other Inflammatory Liver Diseases

Although the role of MADCAM-1/α4β7 in PSC has been widely reported, this theory still lacks comparison with other chronic liver diseases (CLD), Jonathon J Graham et al. found that hepatic expression of MAdCAM-1 was up-regulated in all CLD, including PSC, primary biliary cholangitis (PBC), alcoholic liver disease (ASH), NASH, and Viral hepatitis C (HCV), and that the frequency of hepatic T-cells expressing α4β7 was increased, as compared to normal liver, suggesting that aberrant hepatic recruitment of T-cells of intestinal origin is not unique to PSC exclusively [66].

Increased infiltration of α4β7+ CD4+ T cells and increased MAdCAM-1 expression in the liver and intestines of NASH mice. Treatment of mice with a highly specific neutralizing monoclonal antibody against α4β7 inhibited α4β7+ CD4+ T cell recruitment in the colon and liver of NASH mice, attenuated hepatic and colonic mucosal inflammation, and protected the colonic epithelial barrier function to improve NASH. Similarly, MAdCAM-1, Itga4, and Itgb7 expression was higher in liver tissues of NASH patients than in controls [67]. However, Hannah K. Drescher et al. found that unlike the protective effect of MAdCAM-1 deficiency, β7 integrin deficiency triggered stronger hepatic immune cell infiltration and subsequent hepatic inflammation, promoting NASH and fibrosis progression [68]. This discrepancy can be partially attributed to the fact that complete deficiency of β7 integrin impedes the migration of regulatory T cells to the liver, thereby weakening the suppression of effector T cell proliferation and promoting the secretion of inflammatory cytokines and chemokines. Furthermore, this enhanced inflammatory microenvironment recruits more neutrophils to the liver.

MAdCAM-1 was expressed in tissue sections from 27/28 patients with cirrhosis, mainly localized in the endothelium of the peribiliary blood vessel plexus and in interstitial regions within lymphoid aggregates, and there was also a significant upregulation of *MAdCAM-1* mRNA levels compared to the control group, which was consistent with immunohistochemical analysis [69]. Infiltration of α4β7+ T cells in liver tissue sections was significantly higher in 10 NASH-related and 10 alcoholic steatohepatitis-related cirrhosis patients than in control patients. Mice with CCL4-induced cirrhosis also exhibited greater aggregation of α4β7+ T cells and higher expression of Itga4, Itgb7, and MADCAM-1 [70]. A comprehensive phenotypic and functional characterization of CD8+ T cells in primary biliary cirrhosis concluded that CD45RO^high^ CD57+ CD8^high^ T cells are a subpopulation of cytotoxic memory cells that play a key role in the destruction of bile duct epithelial cells in PBC and that these cells have a higher α4β7 expression compared to other CD8^high^ T cells [71].

Increased expression of MAdCAM-1 in liver tissue was shown in mice with immune hepatitis induced by Concanavalin A (ConA). In contrast, MAdCAM-1-deficient and β7-deficient mice exhibited reduced production of pro-inflammatory mediators and showed protection against ConA-induced liver injury. Additionally, lymphocytes isolated from lymph nodes of wild-type and β7-deficient mice were transferred intravenously into recipient mice lacking mature lymphocytes. The results showed that recipient mice receiving lymphocytes from wild-type mice had more apoptotic hepatocytes and higher expression of MAdCAM-1, indicating that β7 is involved in ConA-induced liver injury and plays a key role in MAdCAM-1 expression [72]. MAdCAM-1 has been reported as one of the nine groups of susceptibility loci shared by inflammatory bowel disease and autoimmune liver disease [73].

As early as 1999, Hillan KJ et al. found that 20 out of 30 patients recruited with HCV showed MAdCAM-1 positivity in liver tissue, with a significant correlation between its expression and the hepatic activity index (HAI) classification [64].

#### 4.2.3. Acute Liver Injury

Acute liver failure (ALF) mice exhibited elevated serum MAdCAM-1 levels that were significantly positively correlated with ALT, an indicator of liver injury. Similarly, serum MAdCAM-1 levels appeared to be significantly elevated in patients with liver failure and were suggested to be a promising early predictor of liver failure. Hepatic tissue *MAdCAM-1* mRNA levels also appeared to be significantly increased, and there was a significant positive correlation with hepatic tissue pro-inflammatory markers, such as TNFα and IL6. Accordingly, acute liver failure mice showed accumulation of α4β7+ CD4+ T cells in the liver [74].

Taken together, this suggests that the role of MADCAM-1/α4β7 in hepatic inflammation is broad and not limited to primary sclerosing cholangitis (Table 1).

### 4.3. Lymphocyte Migration-Associated Intestinal Microbiota and Metabolites

The role of intestinal microbiota and its metabolites in the gut-liver axis is well established and it has been reported that the expression of MAdCAM-1/α4β7 is associated with specific intestinal microbiota and metabolites, especially with bile acids. Conrad Rauber and Guido Kroemer et al. of the Gustave Roussy Cancer Center in France found that after receiving antibiotic treatment, the *Enterocloster* spp. colonizes the intestines of mice and down-regulates MAdCAM-1 expression in PPs and MLN through the accumulation of its metabolites lithocholic acid (LCA) and ursodeoxycholic acid (UDCA), which in turn triggers an exodus of immunosuppressive α4β7+ CD4+ Treg17 cells from the intestine to tumor-draining lymph nodes, aiding in tumor immune escape and thus diminishing the efficacy of the immune checkpoint inhibitor (ICB). And suggests that serum sMAdCAM-1 is a surrogate marker for intestinal dysbiosis. It is noteworthy that exposure to either *Enterocloster* spp., UDCA, or LCA significantly reduced the fluorescence intensity of GFP under the control of the *Madcam1* promoter in sinusoidal endothelial cells (TSEC) [45]. *Lactobacillus plantarum* treatment ameliorated histological damage and decreased the expression of adhesion molecules MAdCAM-1, ICAM-1, and α4β7 in IL10^−/−^ colitis mice [75]. Recipient mice receiving a high-fat diet or low-fiber dietary microbiota exhibited increased expression of MAdCAM-1 in aortic sinus plaques as well as an increase in β7-positive cells compared to controls [76]. Bing Han, Xiaodan Lv et al. in addition to demonstrating the ameliorative effect of Vedolizumab in the mouse model of 2,4,6-trinitrobenzenesulfonic acid-induced colitis, found that baseline intestinal microbiota differed at all levels between the Vedolizumab-responsive group and the non-responsive group. And it was further found by antibiotic depletion of intestinal microbiota experiments that the depletion of microbiota resulted in partial attenuation of the beneficial effects of anti-α4β7 integrins, including weight gain, reduction in DAI scores, and reversal of severe pathologic symptoms. And recipient mice receiving fecal bacteria from Vedolizumab-responsive patients were found to exhibit elevated body weight, improved pathology, and reduced intestinal inflammation through fecal transplantation experiments. In addition, the metabolite clustering of mice in the Vedolizumab-responsive and non-responsive groups was significantly differentiated, with higher levels of bile acids such as chenodeoxycholic acid (CDCA), LCA, and Taurochenodeoxycholic acid (TCDCA) in the responding group. Moreover, CDCA, a recognized FXR agonist, showed significantly higher expression of both FXR and FXR target gene FGF15 in the responding group than in the non-responding group [77]. The association of UDCA with MAdCAM-1 was also validated in in vivo and in vitro experiments. UDCA reduced TNF-α-induced MAdCAM-1 expression in TSEC and brain endothelial cells (bEnd.3) in a dose-dependent manner. Similarly, a significant reduction in MAdCAM-1 expression was seen with the administration of UCDA in MDR2^−/−^/DSS, an animal model mimicking human PSC-IBD [78]. In addition to bile acids, cysteamine is the metabolite with the highest ability to induce functional MAdCAM-1 expression on the hepatic endothelium among VAP-1 substrates [34]. Furthermore, Treg cells were increased in the colon, MLN, and PPs in mice treated with butyric acid, while Treg expressing α4β7, CCR9, and GPR15 were increased in pancreatic lymph nodes (PLN) and pancreas suggesting that intestinal-activated Treg cells migrate to the pancreas and have the function of restoring immune tolerance and delaying diabetic onset during Type 1 Diabetes [79].

Other metabolites also have a role in lymphocyte migration. Short-chain fatty acids (SCFA) increase the expression and mRNA levels of *L-selectin* on neutrophil surfaces. Both propionic acid and butyric acid among SCFA promote neutrophil migration in vitro and in vivo [80]. Compared to the control group, the medium-chain fatty acid octanoic acid increased the expression of L-selectin on T lymphocytes, decreased the proportion of free-flowing T cells, and increased the proportion of rolling lymphocytes compared to the control group. However, it had no significant effect on cell adhesion or migration rate [81]. Administration of propionic acid significantly reduced the migration of Th1 cells from the colon to the spleen in experimental autoimmune uveitis (EAU) to stabilize the subclinical intestinal changes that occur in EAU. Propionic acid may induce Th17 retention in the ileum prior to the onset of uveitis, increasing IL-17 production in the ileum and decreasing the number of Th17 cells in the MLN [82]. In addition, the gut microbiota may also regulate ILC migration and localization through local production of metabolites. The GPR109A receptor on ILC3 senses higher levels of butyric acid in ileal PPs compared to jejunal PPs, promoting ILC3 localization in jejunal PPs [83]. In addition to butyric acid, ILC3 migration is also affected by 7α,25-dihydroxycholesterol and tryptophan, and deletion of 7α,25-OHC prevents ILC3 from localizing to crypt plaques (CPs) in the colon and small bowel, and 7α,25-OHC also mediates the localization of ILC3 to the lymphoid follicles (ILF) and interfollicular regions within the MLN. Increased production of 7α, 25-OHC in inflammatory states promotes GPR183+ ILC3 migration to colonic inflammation sites [84,85]. Tryptophan may enhance ILC3 migration through Ahr regulation of CCR6 [86].

5-Hydroxyindoleacetic acid (5HIAA) and its receptor GPR35 promote neutrophil aggregation to inflamed peritoneum, lymph nodes, skin and other sites. And 5HIAA is required for the induction of neutrophil aggregation [87]. Sodium lactate inhibits CD4+ T cell motility by interfering with glycolysis required for T cell migration, whereas lactate inhibits CD8+ T cells. This effect on different T cell subsets is mediated by different transporter proteins [88]. Migration of sarcosine-treated dendritic cells to lymph nodes and spleen was significantly increased after intradermal injection in the inguinal region of mice. Sarcosine also significantly increased the migration of human and mouse dendritic cells and the expression of CXCR2, CXCL3, and CXCL1 in in vitro experiments. Sarcosine-induced migration was blocked by CXCR2-neutralizing antibodies [89]. Prostaglandin E2 (PGE2) induces dendritic cells to express the lymphoid homing chemokine receptor CCR7 and promotes their migration. Neutrophils treated with pneumococcal haemolysin or Pseudomonas aeruginosa enhance PGE2 production, impeding neutrophil activation and migration, thereby inhibiting an effective immune response [90]. It is concluded in Table 2.

### 4.4. Others

In addition, other integrins [91] also play important roles in inducing lymphocyte migration in response to inflammatory signals and in the pathogenesis of inflammatory enterohepatic diseases [92]. Examples include αLβ2 (LFA-1) and its ligand ICAM-1. The therapeutic agent efalizumab, which blocks this interaction, improves dermatopathological features of psoriasis by decreasing recruitment of inflammatory cells at psoriatic lesions [93,94]. Lifitegrast, a novel small-molecule antagonist that blocks ICAM-1 binding to αLβ2, reduces corneal inflammation and is approved in the United States for the treatment of dry eye [95]. In terms of intestinal inflammation, Pavlick et al. also found that RAG-1^−/−^ recipient mice receiving LFA-1^−/−^ donor T cells showed less evidence of colitis than recipient mice receiving wild-type mouse T cells [96]. The use of an antibody that blocks the integrin MAC-1, which is formed from the β2 subunit and αM, also inhibits inflammatory cell recruitment, reduces ulcer area and number of ulcers, and ameliorates tissue damage in rats with colitis [97]. In addition to leukocyte adhesion integrins, the αv subunit-containing αvβ3 and αvβ5 of the RGD-binding integrins bind to bone bridging proteins in the livers of NAFLD mice, which inhibits autophagosome-lysosome fusion and promotes lipid accumulation. Free fatty acids-induced autophagic damage was attenuated and lipid accumulation was reduced in HepG2 cells using αvβ3 and αvβ5 antibodies [98]. Deletion of the gene encoding the αv integrin subunit effectively targets myofibroblasts in multiple organs and is protective in multiple fibrosis models. Blockade of αv-containing integrins by the small molecule CWHM 12 attenuated liver and lung fibrosis [99]. αvβ3 expressed on stellate cells also has a clear role in fibrosis in NASH, and the use of an antibody that blocks integrin αvβ3 reduces the expression of laminin in high glucose-induced human liver sinusoidal endothelial cells [100]. Expression of αvβ3 detected using the 18F-FPP-RGD2 PET probe in NASH mouse model is strongly associated with histological fibrosis [101,102]. In addition, the use of anti-Itgb1 antibody attenuated pro-inflammatory monocyte infiltration, suppressed hepatic inflammation, and ameliorated hepatic injury and fibrosis in the livers of mice fed a high fat, fructose, and cholesterol diet [103].

**Table 2:** Role of intestinal microbiota or metabolites on lymphocyte migration.

Intestinal Microbiota/Metabolites	Cells	Mechanisms	Refs.
propanoic acid	Th1/Th17 cells	Propionate reduces migration of Th1 cells from the colon to the spleen in experimental EAU and induces Th17 retention in the ileum prior to the onset of uveitis	[82]
SCFA	neutrophil	SCFA promote L-selectin expression on neutrophils to stimulate their migration	[80]
butyric acid	Treg cell	Butyric acid promotes migration of gut-activated Treg cells (expressing α4β7, CCR9 and GPR15) to the pancreas and PLN	[79]
butyric acid	ILC3	Butyric acid regulates the localization of NKp46+ ILC3 in Peyer’s patch	[83]
7α,25-Dihydroxycholesterol(7α,25-OHC)	ILC3	7α,25-OHC and its receptor GPR183 mediate localization of CCR6+ LTi-like ILC3 to crypts, isolated lymphoid follicles	[84,85]
tryptophan	ILC3	Tryptophan may be able to enhance ILC3 migration through Ahr regulation of CCR6	[86]
5-Hydroxyindoleacetic acid (5HIAA)	neutrophil	5HIAA and its receptor GPR35 promote neutrophil aggregation to inflamed peritoneum, lymph nodes, skin and other sites	[87]
lactic acid/ sodium lactate	T cell	Sodium lactate inhibits CD4+ T cell motility and lactate inhibits CD8+ T cell motility	[88]
sarcosine	dendritic cell	Sarcosine increases migration of mouse and human dendritic cells through the CXC chemokine pathway	[89]
ProstaglandinE2 (PGE2)	dendritic cell/ neutrophil	PGE2 induces dendritic cells expressing CCR7 to migrate to lymph nodes, inhibiting neutrophil activation and migration	[90]
caprylic acid	T cell	caprylic acid increases L-selectin expression to stimulate lymphocyte rolling but does not affect lymphocyte adhesion	[81]
*Enterocloster* LCA, UDCA	Treg17 cell	*Enterocloster* down-regulates MAdCAM-1 expression in PPs and MLN through accumulation of its metabolites LCA and UDCA, which in turn causes α4β7+ CD4+ Treg17 cells to leave the intestine and metastasize into tumor-draining lymph nodes	[45]
*Lactobacillus plantarum*	/	*Lactobacillus plantarum* reduces MAdCAM-1, ICAM-1 and α4β7 expression and ameliorates histological damage in IL10^−/−^ colitis mice	[75]
UDCA	/	UDCA inhibits MAdCAM-1 expression in TNF-α-induced TSEC, bEnd.3 cells and MDR2^−/−^/DSS animal model	[78]
cysteamine	/	Deamidation of cysteamine by VAP-1 induces up-regulation of MAdCAM-1 on hepatic endothelium	[34]

## 5. α4β7/MAdCAM-1 as a Drug Target in Inflammatory Enterohepatic Diseases

**Figure 4:**
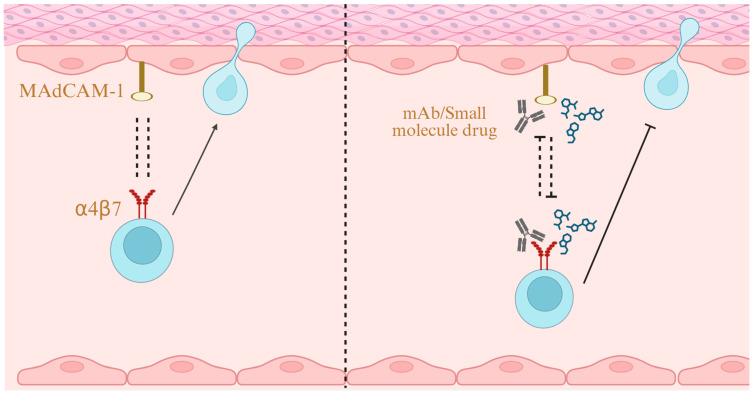
**α4β7/MAdCAM-1 as a Drug Target.** Blocking the interaction between MAdCAM-1 and α4β7 with monoclonal antibodies or small-molecule drugs inhibits lymphocyte migration, thereby ameliorating inflammation. Created in BioRender. Yu, C. (2025) https://BioRender.com/y44ph22 (accessed on 24 October 2025).

A number of therapeutic agents targeting the MAdCAM-1/α4β7 interaction (Figure 4) have been approved or are under investigation in various clinical trial phases.

### 5.1. Monoclonal Antibody

On May 20, 2014, vedolizumab [Entyvio (US, Europe)], a humanized monoclonal antibody against α4β7 integrin, received its first global approval in the United States. Vedolizumab is used for the treatment of adult patients with moderately to severely active ulcerative colitis and Crohn’s disease who have had an inadequate response, loss of response, or intolerance to TNF-a inhibitors or immunomodulators, or who have had an inadequate response or intolerance to, or have developed dependence on corticosteroids [56]. In ulcerative colitis, Vedolizumab was used to induce and maintain clinical response and clinical remission. No significant difference in adverse event incidence was observed between the Vedolizumab and placebo groups [104]. Although adverse events were more common with Vedolizumab for the treatment of Crohn’s disease, patients with active Crohn’s disease treated with Vedolizumab were more likely to achieve remission at week 6 compared to those treated with placebo. Patients who responded to induction therapy were more likely to achieve remission at week 52 if they continued to receive Vedolizumab, compared to those who switched to placebo [105]. In addition to their role in IBD, Kate D. Lynch, Roger W. Chapman et al. found no definitive biochemical response to Vedolizumab in a cohort with primary sclerosing cholangitis and inflammatory bowel disease who had received at least three doses. However, a 20% reduction in alkaline phosphatase (ALP) was observed in some patients, and those with cirrhosis and higher baseline ALP levels were more likely to show a reduction in ALP [106]. This result may be due to the indirect therapeutic effect of vedolizumab, as it has been shown to produce only gut-specific anti-inflammatory activity [57].

Natalizumab, a recombinant humanized monoclonal antibody against the α4 integrin, was the first drug approved for the treatment of Crohn’s disease, increasing clinical remission and response rates and improving quality of life [107]. However, Natalizumab has been limited due to the fact that 1 in 1000 treated individuals may develop progressive multifocal leukoencephalopathy [108]. Abrilumab, a fully human monoclonal IgG2 antibody against α4β7, demonstrated significantly better remission rates after treatment in both Phase II studies against moderate to severe CD and UC than in the placebo group [109]. However, no Phase III clinical trial enrollment information is available to date. A phase III, multicenter, double-blind, placebo-controlled study of Etrolizumab, a monoclonal antibody specifically targeting the β7 subunit, in patients with moderately-to-severely active ulcerative colitis who had been previously treated with anti-TNF drugs, showed that the Etrolizumab group was able to achieve a higher rate of remission compared to the control group. However, no significant difference was observed between the groups on the primary maintenance endpoint of remission at week 66 [110]. In HIBISCUS I and HIBISCUS II, two identically designed, multicenter, phase III, randomized, double-blind, placebo-controlled and active-controlled studies, Etrastuzumab given to patients with moderately to severely active ulcerative colitis who had not been treated with an anti-TNF agent, Etrastuzumab was superior to placebo in inducing remission in HIBISCUS I, but its performance was not superior to Adalimumab in both HIBISCUS I and HIBISCUS II trials [111], and it also performed similarly to Infliximab (tumor necrosis factor inhibitor) [112]. Ontamalimab, a fully human immunoglobulin G2 monoclonal antibody against MAdCAM-1, was shown to achieve the primary endpoint of clinical remission in more patients than the placebo group. It also showed a significant advantage in endoscopic improvement with no safety concerns [113].

### 5.2. Small Molecule Drug

MORF-057, an orally administered small molecule inhibitor designed to selectively inhibit integrin α4β7, is currently undergoing its evaluation in adult patients with moderately to severely active ulcerative colitis and Crohn’s disease [114,115]. AJM300, an oral α4 integrin antagonist, induced clinical responses in patients with moderately active ulcerative colitis and in patients with inadequate response or intolerance to Mesalazine, and there was no difference in the incidence of adverse events compared to controls [116]. It was first approved for the treatment of patients with moderate ulcerative colitis who were not responding well to treatment with 5-aminosalicylic acid in Japan in 2022 [117]. Although the oral α4β7 antagonist peptide PTG-100 initially failed to meet the primary endpoint in the phase II study, blinded re-readings of endoscopies from 65 participants evaluated for futility showed that the trial would not reach futility, and colon biopsy results suggested that PTG-100 could improve histology and that the trial could continue as planned [118]. The key clinical trial outcomes of these drugs are summarized in Table 3, while their current developmental status is presented in Table 4.

## 6. Conclusions and Perspective

The recruitment and migration of lymphocytes are central to the immune response, enabling their precise homing to sites of inflammation. This process plays a critical role in maintaining immune homeostasis, initiating specific immune reactions, and regulating inflammatory progression. Furthermore, it is also highly regulated and requires coordination by adhesion molecules expressed on homing lymphocytes and corresponding ligands expressed by endothelial cells, in which MAdCAM-1/α4β7 has an important role in the regulatory process [121]. Excessive lymphocyte migration, triggered by dysregulated MAdCAM-1/α4β7 expression, not only disrupts intestinal immune balance and drives local inflammation but also mediates the spread of inflammation to the liver through the gut-liver axis. This sequential process constitutes the core pathological feature of inflammatory enterohepatic diseases, highlighting the integral link between gut and liver in inflammatory mechanisms. Therefore, inhibiting aberrant MAdCAM-1/α4β7 binding is essential for curbing this connected series of intestinal and hepatic inflammatory events [122].

Blocking lymphocyte migration to the liver may be a potential mechanism to reduce inflammation and prevent disease progression. Administration of both MAdCAM-1 mAb and α4β7 mAb to Western diet-induced NASH mice ameliorated their inflammation and liver fibrosis [67]. Similarly, the use of MAdCAM-1 mAb and α4β7 mAb resulted in reduced hepatic inflammation and blocked the progression of fibrosis in mice with CCL4-induced hepatic fibrosis [70]. The lack of MAdCAM-1 or β7 integrins also reduced pro-inflammatory mediator production and attenuated ConA-induced liver injury [72]. However, Drescher et al. also found that β7 integrin deficiency triggered more intense hepatic immune cell infiltration and subsequent hepatic inflammation. This discrepancy can be partially explained by key methodological differences between the present study and the work by Rai et al. Specifically, Drescher et al. utilized β7 integrin-deficient mice, whereas Rai et al. employed a neutralizing monoclonal antibody against α4β7 to block its interaction with MAdCAM-1. The complete absence of β7 integrin impaired the migration of regulatory T cells to the liver, thereby attenuating the suppression of effector T cell proliferation and promoting the secretion of inflammatory cytokines and chemokines. This enhanced inflammatory milieu recruited more neutrophils to the liver. Moreover, β7^−^/^−^ mice exhibited elevated levels of IL-17 mRNA, a cytokine produced by Th17 cells that is known to promote neutrophil recruitment. More directly, neutrophils do not express integrin α4β7, indicating that their migration to inflammatory sites is independent of β7 [68]. And this controversy may require further research. Moreover, in clinical trials, Vedolizumab did not significantly improve the levels of biochemical markers such as hepatic transaminases and bilirubin in patients with PSC, but the 20% reduction in ALP was observed in some patients, and patients with cirrhosis and higher ALP at baseline were more likely to respond with a reduction in ALP [106,123]. This may be related to the fact that vedolizumab, by blocking α4β7 integrin, exclusively produces gut-specific anti-inflammatory activity. In healthy non-human primates treated with vedolizumab, a significant decrease in the frequency of β7+ lymphocytes in gastrointestinal tissues and a significant increase in the frequency of α4β7+ memory helper T lymphocytes in peripheral blood were observed. Additionally, Vedolizumab did not suppress systemic adaptive or innate immune responses. These data suggest that Vedolizumab has a gut-specific anti-inflammatory effect [124]. Moreover, a vaccination study in healthy volunteers showed that vedolizumab attenuated the response to enteral antigen challenge (oral cholera vaccine) without affecting the response to parenteral antigen challenge (intramuscular hepatitis B vaccine), indicating a high degree of gut-specific anti-inflammatory activity for Vedolizumab [57]. In a limited cohort, all three patients with comorbid CD and cirrhosis, who had undergone ileal or colonic resection, or who were unresponsive to treatments such as infliximab, were well tolerated by vedolizumab in the present study, and none developed significant infectious complications or decompensated cirrhosis [125].

The current gut selectivity of drugs is likely attributable to the high constitutive expression of MAdCAM-1 in the gut, compared to its minimal expression in the liver under physiological conditions. However, hepatic MAdCAM-1 is upregulated during immune activation. Therefore, future therapeutic strategies for liver inflammatory diseases may focus on blocking the migration of α4β7-expressing cells to the liver. One such strategy could involve the construction of GalNAc-modified, ROS-responsive polymeric nanoparticles to encapsulate α4β7/MAdCAM-1 antagonists [126,127]. Such a targeted delivery system could enhance liver targeting while reducing systemic immunosuppression. Combination strategies with agents that address other hepatic pathological features, such as disordered lipid metabolism, should also be considered. Furthermore, given that combination therapies using microecological modulators and small-molecule drugs have been previously documented [128], the potential synergistic effect between anti- MAdCAM-1/α4β7 therapy and FXR agonists—such as obeticholic acid—is highly promising. Such a combination strategy could simultaneously inhibit the infiltration of aberrant immune cells into inflamed tissues and restore microbiome-metabolite homeostasis, thereby addressing both the inflammatory and metabolic pathological features of the disease. More importantly, to rigorously test this hypothesis in humans, well-designed Phase II/I trials in PSC and NASH should be implemented. These trials ought to incorporate appropriate and stable biomarkers for efficacy evaluation and adopt stratified randomization designs to enable further assessment in specific patient subpopulations. Beyond therapeutic applications, specific molecular imaging probes such as 18F-FPP-RGD2 enable non-invasive, in vivo, quantitative detection of specific molecular pathway activity underlying diseases. These probes can be used not only for diagnosis and staging but also for real-time treatment guidance and monitoring. Additionally, as highlighted in the study by Marine Fidelle et al., serum MAdCAM-1 may serve as a strong prognostic indicator of treatment response in cancer patients [45], raising the possibility of using specific MAdCAM-1 detection for diagnosing or monitoring treatment of inflammatory enterohepatic diseases. All these aspects warrant further research and exploration.

In addition to the diseases mentioned above, gut-associated lymphoid tissues have been shown to be a key site of human immunodeficiency virus (HIV) viral replication, especially during the acute phase of HIV infection. The doping of host cell proteins by HIV virosomes has long been recognized, and α4β7 remains functionally active after its doping, with the ability to bind to MAdCAM-1, and may contribute to early infection of intestinal tissues through binding to MAdCAM-1 [129]. Thus, antibodies that block viral admixture of α4β7 may ameliorate intestinal damage and slow disease progression during early HIV infection [130]. In addition, drugs targeting α4 are being experimented and explored in diseases such as multiple sclerosis, asthma and allergic conjunctivitis [131,132,133,134,135]. The role of MAdCAM-1/α4β7 in inflammatory diseases is widely recognized.

Currently, several emerging drugs targeting MAdCAM-1/α4β7 to block lymphocyte homing primarily focus on IBD; however, their potential role in liver inflammation remains largely unexplored and warrants investigation. This review summarizes the current state of development, key research findings, and existing challenges, aiming to facilitate the discovery and clinical development of molecules or drugs targeting MAdCAM-1/α4β7 that could benefit liver inflammation.

## Figures and Tables

**Table 1:** Role of MAdCAM-1/α4β7 in inflammatory liver disease.

Diseases	Cohort/Model	Main Effects	Ref.
PSC	5 patients with PSC	Antibodies against MAdCAM-1 (10G3), α4β7 (ACT-1) blocked the adhesion of peripheral blood lymphocytes to MAdCAM-1-transfected CHO cells, to the hepatic portal vein, and the hepatic blood sinusoids	[63]
	16 patients with PSC who underwent liver transplantation	14/16 cases with positive immunohistochemical MAdCAM-1 staining	[63]
	Liver biopsy of 9 patients with PSC	α4β7+ T lymphocyte infiltration was significantly higher than in patients with non-inflammatory liver disease	[62]
	5 patients with PSC who underwent liver transplantation	5/5 cases with positive immunohistochemical MAdCAM-1 staining	[64]
	Liver biopsy of 7 patients with Long-term PSC	The H-score of immunohistochemical MAdCAM-1 staining was significantly higher than that of the control. The proportion of β7-positive T cells in the liver was increased and positively correlated with PSC disease duration	[65]
NASH	Western diet-fed F11r^−/−^ mice were administered mAbs targeting α4β7 and MAdCAM-1	Amelioration of hepatic inflammation and fibrosis observed in histopathology. Significant reduction in serum ALT and AST levels, inflammatory factors such as *TNF-α*, and liver fibrosis indicators such as *Acta2*.	[67]
	Western diet-fed F11r^−/−^ mice	α4β7+ CD4+ T cells exhibited increased accumulation in peripheral blood, Peyer’s patches, colonic lamina propria, and liver tissues, accompanied by elevated MAdCAM-1 expression in both colon and liver compared to WT mice	[67]
	Liver biopsy of 5 patients with NASH	Immunohistochemical analysis revealed a larger MAdCAM-1-positive area in the livers of NASH patients compared to controls, with significant upregulation of *MAdCAM-1*, *ITGA4*, and *ITGB7* mRNA levels	[67]
	MAdCAM-1-deficient mice were fed high-fat diet or methionine-choline deficient diet	MAdCAM-1 deficiency ameliorates liver injury and fibrosis by enhancing antioxidant and anti-inflammatory immune response	[68]
Cirrhosis	CCL4-induced mice were administered mAbs targeting α4β7 and MAdCAM-1	Protected against liver injury and inhibited the progression of fibrosis, reduced serum ALT, AST levels, the positive staining of Sirius red, and the expression of liver fibrosis indicator *Col1a2*, and so on	[70]
	CCL4-induced mice	α4β7+ CD4+ T cells were markedly accumulated In the liver tissues, accompanied by enhanced immunopositivity of MAdCAM-1 and α4β7 compared to WT mice. The mRNA levels of *MAdCAM-1*, *ITGA4*, and *ITGB7* were significantly upregulated	[70]
	10 patients with NASH-related and 10 patients with ASH-related cirrhosis who underwent liver transplantation	Stronger immunopositivity for α4β7 compared to controls	[70]
	28 patients with cirrhosis who underwent liver transplantation	27/28 cases with positive immunohistochemical MAdCAM-1 staining, with significant upregulation of *MAdCAM-1* mRNA level	[69]
	24 patients with PBC	The frequency of CD45RO^high^ CD57+ CD8^high^ subpopulations expressing α4β7 in the peripheral blood of PBC patients was significantly higher than that of controls	[71]
	Liver biopsy of 21 patients with PBC and 5 patients with PBC who underwent liver transplantation	15/21 cases with positive immunohistochemical MAdCAM-1 staining, 5/5 cases with positive immunohistochemical MAdCAM-1 staining	[64]
	11 patients with PBC who underwent liver transplantation	7/11 cases with positive immunohistochemical MAdCAM-1 staining	[63]
Acute Immune-Mediated Hepatitis	ConA-induced mice	The area of positive immunofluorescence staining and mRNA level of MAdCAM-1 in liver tissue were significantly higher than those in WT mice	[72]
	ConA-induced MAdCAM-1^−/−^ and β7^−/−^ mice	Reducing the production of pro-inflammatory mediators, such as IFN-γ, resulted in liver tissue necrosis, hepatocyte apoptosis, and serum ALT levels, which were significantly lower than those of WT mice after receiving ConA	[72]
ALF	LPS/D-GalN-induced mice	MAdCAM-1 mRNA and protein levels in serum and liver tissue were significantly higher than those in WT mice. Higher aggregates ofα4β7+ CD4+ T cells in liver tissue of ALF mice.	[74]
	12 patients with ALF	Exhibited a significantly higher level of serum MAdCAM1, more area of MAdCAM-1, β7 positive staining in liver tissue compared with healthy controls	[74]
HCV	Liver biopsy of 30 patients with HCV	20/30 cases with positive immunohistochemical MAdCAM-1 staining	[64]
Autoimmune hepatitis	10 patients with autoimmune hepatitis who underwent liver transplantation	7/10 cases with positive immunohistochemical MAdCAM-1 staining	[63]

**Table 3:** Clinical trial results of MAdCAM-1/α4β7-targeted therapies.

Drug Name	Disease	Efficacy	Safety	Limitations
Natalizumab	CD	Primary endpoint (clinical remission at Week 6) was not met; however, positive signals were observed at multiple time points	Generally well-tolerated	The short-term (12-week) study design precluded assessment of long-term risks, such as progressive multifocal leukoencephalopathy
Vedolizumab	UC	Clinical response significantly higher at Wk6 (47.1% vs. 25.5%); remission at Wk52 significantly higher (41.8%/44.8% vs. 15.9%)	Generally well-tolerated	Optimal induction duration and minimum effective dose not determined
	CD	Significantly higher clinical remission vs. placebo at Wk6 (14.5% vs. 6.8%) and Wk52 (39.0%/36.4% vs. 21.6%) in maintenance responders	Higher incidence of serious AEs (24.4% vs. 15.3%)	Optimal patient population not defined; combination therapy effects not assessed
	PSC	Biochemical response in a subset of patients (20.6%); effective for concomitant IBD	Significant liver biochemical worsening in some patients	Retrospective design; lack of control group; UDCA use post-baseline unclear
Etrolizumab	UC	Significant induction of remission at Wk 14 (18.5% vs. 6.3%)	Generally well-tolerated	Lack of statistically significant efficacy in the maintenance phase
	CD	Significant improvement in clinical remission (35% vs. 24%) and endoscopic improvement (24% vs. 12%) at Week 66 vs. placebo. No significant benefit during induction	Generally well-tolerated	mITT analysis; potentially underestimated placebo response; unusually long study duration; premature closure of induction cohort 3
Abrilumab	UC	Unadjusted remission rates at Week 8 were significantly higher with abrilumab (13.3%, 12.7%) than placebo (4.3%)	Generally well-tolerated	Dosing errors; short-term study; limited subgroup analysis by prior TNF antagonist exposure
Ontamalimab	UC	Significantly higher clinical remission vs. placebo at Wk 12 (29.8%/29.5% vs. 15.8%/12.5%) and Wk 52 (53.5%/40.2% vs. 8.2%/12.8%)	Generally well-tolerated	Clinical development program prematurely discontinued
AJM300	UC	Clinical response rate significantly higher with AJM300 vs. placebo at Week 8 (45% vs. 21%)	Generally well-tolerated	Limited sample size/retreatment cycles; severe/refractory UC populations not evaluated; open-label retreatment phase
PTG-100	UC	Primary endpoint (remission at Wk12) not met initially	Generally well-tolerated	limited sample size; initial primary endpoint failure; results interpreted with caution.
MORF-057	UC	At Week 12, mean reduction from baseline in RHI score was −6.4; 22.9% of participants achieved RHI remission (score ≤ 3)	Generally well-tolerated	Small sample size; high dropout rate; lack of placebo control

**Table 4:** Current development status of MAdCAM-1/α4β7-targeted therapies.

Drug Name	Mechanism of Action	Disease	Approval/ Phase	Primary Developer/ Company	Source
Natalizumab	Humanized IgG4 anti-α4 integrin subunit mAb	CD	Licensed in USA	Biogen Idec	https://www.accessdata.fda.gov/scripts/cder/daf/index.cfm?event=overview.process&ApplNo=125104 [119] (accessed on 19 February 2025)
Vedolizumab	Humanized IgG1 anti-α4β7 integrin dimer mAb	CD/UC	Licensed	Takeda	https://www.accessdata.fda.gov/scripts/cder/daf/index.cfm?event=overview.process&ApplNo=125476 [120] (accessed on 19 February 2025)
Etrolizumab	Humanized IgG1 anti-β7 integrin subunit mAb	UC/CD	Phase III	Roche	UC: NCT02165215 NCT02100696 CD: NCT02394028
Abrilumab	Human IgG2 anti-α4β7 integrin dimer mAb	CD/UC	Phase II	Amgen	CD: NCT01696396 UC: NCT01694485
Ontamalimab	Human IgG2 anti-MAdCMA-1 mAb	UC	Phase III	Takeda	NCT03290781
AJM300	α4 integrin subunit antagonist	UC	Phase III	Meiji Seika Pharma/ EA Pharma	NCT03531892
PTG-100	α4β7 integrin-specific peptide	UC	Phase IIb	Protagonist Therapeutics	NCT02895100
MORF-057	small molecule α4β7 integrin inhibitor	UC/CD	Phase II	Morphic Therapeutic/ Eli Lilly	UC: NCT05291689 NCT05611671 CD: NCT06226883

## Data Availability

Not applicable.

## Abbreviations

IBD	Inflammatory bowel disease
UC	Ulcerative colitis
CD	Crohn’s disease
PSC	Primary sclerosing cholangitis
NASH	Non-alcoholic steatohepatitis
PSI	plexin-semaphorin-integrin
RGD	Arginine-Glycine-Aspartic Acid
IgSF	Immunoglobulin superfamily
MAdCAM-1	Mucosal address in adhesion molecule 1
ICAM	Intercellular adhesion molecule
VCAM-1	Vascular cell adhesion molecule
PECAM-1	Platelet/endothelial cell adhesion molecule
HEV	High endothelial venule
GALT	Gut-associated lymphoid tissues
RAR	retinoic acid receptor
RXR	retinoid X receptor
RAREs	retinoic acid response elements
PPs	Peyer’s patche
MLN	Mesenteric lymph nodes
DSS	Dextran Sulfate Sodium Salt
OVA	Ovalbumin
VAP1	Vascular adhesion protein-1
MA	Methylamine
RA	Retinoic acid
EGID	eosinophilic gastrointestinal diseases
CX3CR1	CX3C chemokine receptor 1
CX3CL1	CX3C chemokine ligand 1
ILCs	Innate lymphoid cells
FDA	Food and Drug Administration
PBC	Primary biliary cirrhosis
CLD	Chronic liver diseases
ASH	Alcoholic steatohepatitis
ALF	Acute liver failure
HCV	Viral hepatitis C
HAI	hepatic activity index
ConA	Concanavalin A
LCA	Lithocholic acid
UDCA	Ursodeoxycholic acid
ICB	immune checkpoint inhibitor
CDCA	Chenodeoxycholic acid
TCDCA	Taurochenodeoxycholic acid
PLN	Pancreatic lymph nodes
SCFA	Short-chain fatty acids
EAU	Experimental autoimmune uveitis
CPs	Crypt plaques
ILF	Lymphoid follicles
5HIAA	5-Hydroxyindoleacetic acid
PGE2	Prostaglandin E2
ALP	Alkaline phosphatase
HIV	human immunodeficiency virus
